# Of Bright’s Disease of the Kidneys

**Published:** 1846-08

**Authors:** G. N. Burwell


### BUFFALO MEDICAL JOURNAL

AND

### MONTHLY REVIEW.

**VOL. 2.** AUGUST, 1846. **NO.3.**

### ORIGINAL COMMUNICATIONS.

ART. I.—*Of Bright's Disease of the Kidneys.* By G. N. Burwell M. D.

In the number of this Journal for June last, I gave a short history of four cases of Anasarca and Ascites, with Albumen in the Urine. I propose in this, and the succeeding number, to continue the subject, by giving a short account of the discovery of the connection of these affections with an albuminous discharge from the kidneys, and a general review of the present condition of our knowledge of the disease.

It has been attempted to be shown that this affection was recognised even as far back as the time of Hippocrates; but of this there must be considerable doubt. The fact of the occasional coagulation of the urine may have been noticed by that physician, but of the connection of this fact with a peculiar organic disease of the kidneys, he could not have known. This connection was not shown until 1827, when the valuable discoveries of Richard Bright, of London, were first published.

The fact that there was an albuminous or serous discharge from the kidneys in some dropsies, was very generally known some thirty years before this publication of Dr. Bright. His merit consists in endeavouring to connect that discharge with a specific organic change in the secreting structure of the kidneys. Before his reseaches, this discharge was connected with dropsy, and not with the organic disease—the dropsy being now considered as much of a symptom as the discharge itself.

The discovery of the occasional existence of albumen in the urine seems to have been accidental. It was first made, as far as I can ascertain, while endeavoring to obtain a saccharine extract from the urine of persons supposed to be labouring under diabetes mellitus, and was first described as a variety of this disease under the name of "mucilagious diabetes." Great attention was paid to the subject of Diabetes during the latter part of the last century. If a patient had poor health, with progressive emaciation, some febrile symptoms, and discharged more urine than natural in twenty-four hours, he was supposed to labour under diabetes; and one of the methods then resorted to of ascertaining whether this was so, was to evaporate his urine to obtain a saccharine extract. While submitting a specimen of urine to this test, "to my great surprise," says Blackall, "when the heat rose to 160° F. the fluid became uniformly opaque and white, and a considerable precipitate took place, which, when strained, but not much dried, amounted in weight to more than half an ounce, from two quarts of urine, or about one ounce from the quantity discharged daily. A similar effect was produced by nitrous acid."

Farther investigations, especially by Cruickshank (and previous to the researches by Blackall) connected this "mucilaginous" discharge with general dropsy, rather than with diabetes. This writer, accordingly, divides dropsies into two kinds, "first, general dropsy, in which the urine coagulates like diluted serum of the blood; and second, dropsy proceeding from unsound viscera, in which the urine is usually high coloured and scanty, deposites on cooling a pink colored sediment, and does not coagulate either by heat, or the nitrous acid."

The next publication of importance, after Cruickshank's, on this subject, were a couple of papers by Dr. Wells, in 1812; one entitled "observations on the Dropsy which succeeds Scarlet Fever:", the other "on the presence of Red matter, and Serum of the blood in the urine of Dropsy which has not originated in Scarlet Fever."

He insists particularly on the suddenness of the accession of anasarca and ascites, after scarlatina; the acuteness and inflammatory nature of the attack, as shown by the frequent occurrence of peritoneal and pleuritic inflammation; and the extreme danger of the patient so early as the third day. In a large proportion of cases he found red blood to be deposited in the urine, and in all but two, and those slight cases, it was coagulable.

Respecting coagulable urine in other diseases, he concludes that urine containing a considerable quantity of serum, very rarely, if at all, occurs in any disease in England, except dropsy; and even in those very rare cases has been induced by mercury. The fact is noticed that the kidneys have been diseased in an unusual proportion of cases where post mortem dissections have been made.

Almost simultaneously with the publication of Dr. Wells' papers, Dr. Blackall's work made its appearance, So important were its observations and precepts considered, that in the course of six or eight years it went through three editions, in London; and in 1820 Prof. Chapman, of the

University of Pennsylvania, estimating it very highly "as exhibiting correct views of the pathology and treatment of Dropsy," caused a republication to be made in this country.

Dr. Blackall follows the important distinctions of Cruickshank, and describes the subject of dropsy under two heads: *first* where the urine is not coagulable by heat; and *second*, where it is thus coagulable.

A period of fifteen years now elapsed without any farther publications upon the subject of marked novelty or interest ; Dr. Bright's first publications then appeared, in which he, for the first one, clearly and distinctly pointed out the relations between the discharge of coagulable urine and a particular form of disorganization of the kidneys. This constitutes the third stage, or epoch, in the history of this disease.

Immediately there appeared a great number of investigators in the field, among whom Drs. Christison and Gregory, of Edinburgh, Dr. Osborne, of Dublin, Drs. Prout, Barlow, and Watson of London, and Rayer, of Paris, rank as the most conspicuous. By their indefatigable industry, the disease was soon investigated in all its relations, as far as the means of observation and chemical analysis they possessed, would allow them to go. This brings us up to 1836 to 1840; to the end of the third epoch.

Since the dates last mentioned, a new set of observers have directed their attention to the elucidation of what remained dark and unknown of the disease; and which promises some interesting results to its pathology, without, however, as yet, adding much to our knowledge of the causes, and their mode of operation in the production of the disease, or to the treatment, except such as necessity follows a correct pathology of any disease—I refer here particulary to the microscopical observers of the disease. Dr. Todd stands at the head of this list. His friend, Dr. Johnson, has also made some interesting experiments and observations. I have not seen any account of microscopical examinations of the disease by the French.

Having thus hurriedly noted some of the more prominent points in the history of our knowledge of this disease, I propose next to point out the principal anatomical characters, causes, symptoms and treatment of that form of dropsy which proceeds from renal disease. For this purpose I prefer rather to avail myself of the labors of the observers during what I call the third epoch in the history of the disease, than to use my own observations made during two and a half years attendance upon the Hospitals of Philadelphia. These but assure me of the correctness of their facts, (most of which I have seen confirmed time and again) without affording me anything new respecting the history or treatment of the disease.

I purposely make no reference to the observations of the microscopical observers, prefering hereafter to embody them by themselves.

First, as to the name of the disease. The most common one by which it is known is "Bright's disease of the Kidneys," and simply "Bright's Disease," after the author who first described it. Dr. Christison described it under the name of "Granular Degeneration of the kidneys;" Dr. Watson under the head of "Renal Dropsy;" Rayer as "Albuminous Nephritis." No one of these seems to meet with a general concurrence of the Profession, and for the want of a better designation (and the time has not yet come to give it a name expressive of its pathology) I shall describe it under the name first above given.

The true pathological character of Bright's disease seems to be the deposition of an amorphous matter without the vessels; first into the cortical structure, and, resembling lymph more nearly than any other deposit. It is not subject to absorption as are deposits of lymph, nor will it soften down into pus. The term cacoplastic is applied to it by Williams and other pathologists meaning by it an imperfectly organizable matter. It is supposed by them to resemble the deposits about the valves of the heart, and the cicatrices of burns. It has the peculiar contractile property of those cicatrices, which explains the general contraction of the kidneys in the latter stages of the disease, as well as the different partial changes in their form, and the depressions and corrugations of their surface.

If the deposit first take place during the acute form of the disease, it is of a dark red color, often so nearly resembling the surrounding textures, that it connot be well distinguished from them unless the kidney be successfully injected. By this means, says Christison the injecting matter fills the vessels, and marks out and defines the deposit, which, of itself, cannot be injected. It is not often that an opportunity presents itself of examining the kidneys at this stage of the disease. In the more chronic stages the deposit is of a yellowish, or yellowish-white color, and on cutting the kidney open may present the appearance of small yellow granulations interspersed through the cortical substance, or by its abundance giving more of a homogeneous appearance to the organ, occasionally very nearly resembling the section of a parsnip. (*Watson.*)

The extent of the deposit is at first, except in acute cases, generally very limited, being confined, perhaps, to a single pyramid of Ferrein; bnt from this small beginning it almost invariably extends from one portion to another, or new centers of deposit arise, until after some months, or years even, the whole of both kidneys become filled with it, altering their form, and destroying nearly all the healthy secreting structure. It is however rare for both kidneys to be equally diseased.

The action of the deposite is supposed to be purely mechanical; it destroys the vessels about it by pressure, obstructing thereby the blood-vessels, and producing atrophy of the secreting portions. The kidneys are incapacitated only as the deposit increases in extent and number of points. Those parts of these organs to which the deposit has not reached, continue to perform their functions perfectly.

It is a curious and important fact, and one which demonstrates the specific or constitutional nature of the disease, that it is never confined to one kidney. Both always show evidence of it where it exists at all, although not equally.—

Accompanying this deposit we always find various changes in the circulation, size, form, weight, and other physical characters of the kidneys. In primary and acute attacks they are congested, enlarged, increased in weight and diminished in consistence. The size and consistence of the kidney are in most cases inversely proportional to each other. (*Watson.*) There are, of course, various shades of color, according to the degree of the hyperæmia, and whether it partakes more of the arterial or venous form. As the attack varies from the acute to the chronic form, so do these physical properties vary. They are the non-essentials of the pathological characters of the disease, and need not be particularly described in this place. We have no means of diagnosis which after death will enable us to prognosticate the exact degree and kind of these various alterations. We know that the more the disease partakes of the acute form, the more likely we are to find the kidneys enlarged, heavy, friable and congested; and the more chronic it has been in its progress, the more probability there is that they will be found contracted, dense, firm, and irregular in form, from different degrees of contraction of different portions of the deposit; and even complete atrophy of an entire kidney be produced.

These are the two extremes. More frequently will the kidneys be found in what Christison calls the middle stage;—the deposit is here chiefly confined to the cortical portion; the kidney is moderately enlarged, mottled, roughened-externally, while internally (on a section being made) the cortical texture has lost its natural reddish brown hue and shaded appearance, and become of a yellowish grey color, more or less uniform according to the extent of the deposit.

*The causes* may be divided into the constitutional, or predisposing, and the exciting. The predisposing causes are necessarily constitutional, intrinsic but may be hereditary or acquired. We do not know in what the hereditary predisposition exists; but we do know more about the causes which give an acquired predisposition. That which English authors account as the most common in London and Edinburgh, is intemperance; — three-fourths of the cases observed by Dr. Christison being referable to this cause — Dr. Bright alludes to the great influence of the same cause. In France the most frequent predisposing cause is habitual exposure to cold and damp. Intemperance not being so prevalent in Paris, as in the large cities of England, does not, of course, become so prominent a cause. As intemperance and exposure can scarcely be separated, it becomes a question whether the latter ought not to be considered as the principal cause of the predisposition the system acquires, and the former more as an accessory.

Scrofulous and syphilitic disorders, are considered, by many, as powerful predisposing causes. There seems also to be some peculiar connection between diseases of the heart and liver and this form of kidney disease; but their relations have not yet been satisfactorily investigated. It is this combination which has become so well known under the title of the "triple lesion," and although freqnently met with, the exceptions are numerous enough seriously to embarrass observers in their endeavors to discover and establish the connections of the diseases as to their causes and reciprocal effects.

Some statistical observations have been recently published by Dr. Peacock, to shew the frequent co-existence of Bright's disease with phthisis; the former apparently *secondary* to the latter. My own limited experience accords more nearly with that of Dr. Bright, that phthisis is an unusual complication. Rayer and Christison state that the two diseases are frequently combined, while Dr. Watson states that his experience would not have led him to that opinion.—M. Solon, he says, doubts whether the co-existence of pulmonary consumption and this renal malady is more than casual. Farther observations upon this point as well as upon the "triple lesion" are wanted to settle present doubts and contradictions.

Scarlet fever remains to be enumerated among the causes which give a constitutional predisposition to the disease. As this fever occurs so generally among children, it is to them the most frequent cause of Bright's disease. But it is only as a predisposing cause that it acts. Exposure to cold from insufficient clothing or carelessness, at the period of desquamation, is, with a few exceptional cases, the immediate or exciting cause. The symptoms of the disease may shew themselves, according to Dr. Wells, as early as the sixteenth day, or as late as the twenty-fifth day. After the fourth week, when desquamation is completed, he says, it is no longer to be dreaded. There is a peculiarity respecting the cases after this cause which is seldom experienced in cases from other causes. They form the only class of cases where permanent recovery is common, a large proportion of them recovering under proper and prompt treatment. This is so general, and contrasts so greatly with the unfavorable result of the common cases met with, that some authors have doubted whether they ought to be considered as cases of kidney disease. I do not participate in these doubts, *first* because in cases of death in the early stages of dropsy after scarlet fever, the kidneys have been found repeatedly in the early stage of congestion and granulation precisely similar to cases where patients have died of acute anasarca from exposure to cold: *second*, because where death occurs at a later stage, the yellow deposit is generally found; and *third*, because cases are occasionally met with where scarlet fever has without doubt laid the foundation of Dropsy, which has, from the first, taken on the chronic form of the disease, and where after death the kidneys have exhibited the advanced stages of renal degeneration.

It has often been asked why scarlet fever should be the only one of the ex-anthemata which predisposes to this disease, and how does it thus predispose? Authors have generally confessed their ignorance upon these points. The only attempt at explanation is that of Barthez and Rilliet, in their work on diseases of children. "We believe," they say "that it is the seat rather than the intensity or nature of the inflammation which leads to this result. The seat of variola is the cutaneous follicles, that of rubeola the unctous body, or rather the superficial net work; while scarlatina appears to be an inflammation of the sub-cutaneous lymphatic tissue whose functions are at once exhalent and absorbent. This is the cause of the anasarca, for, by reason of the lesion of the lymphatic tissue, the cutaneous exhalation is imperfectly performed, and upon its suppression by the accession of even slight cold, an effusion results. If this occurs in rubeola or variola it is because the cutaneous inflammation is sometimes propagated to the lymphatic tissue." While this offers a plausible answer to the question proposed, it makes this inflammation of the lymphatic tissue of the skin the proximate cause of the anasarca after scarlatina, rather than any disease of the kidneys; and these authors accordingly state that this is the most frequent cause of anasarca in children. In giving this as the result of their observations, they also state that nephritis (Bright's disease,) is the next most frequent cause. It ought to be mentioned here, that the statement of these writers respecting the distinct and separate seats of these exanthemata, have not received confirmation from other observers.

Pregnancy is said sometimes to produce the disease by the womb, during the latter months of gestation, obstructing the renal veins and causing congestion of the kidneys.

Authors are disagreed as to the influence of diseases of the urethra and bladder as predisposing causes. Rayer thinks they have little or no influence in producing albuminous nephritis.

The epidemic constitution of the year is said to dispose to this form of diseased kidney, from the fact that in an epidemic of scarlet fever in Edinburgh, in the winter of 1835–36, one seventh of the cases were followed by anasarca.

Sex and age have an influence. Men are more often attacked than women. It is very rarely met with in early life except as the result of scarlet fever, but it becomes more frequent as age increases, until it reaches its maximum frequency at the age between thirty and forty years. Old age is not exempt. Occupation has an influence only as it necessitates to exposure to the vicissitudes of cold and wet.

The only exciting cause which demands particular notice is exposure to cold, whereby the surface is chilled, and the perspiration checked. The functions of the skin and of the kidneys are to a great extent complemental. Whatever disturbs the former, is liable to affect the latter. With some authors the suppression of the cutaneous circulation and secretion by cold, is considered as the great cause of this form of renal disease, and in their efforts at cure they aim almost solely to procure and keep up a perspirable state of the surface. I have already spoken of cold as a predisposing cause. I speak of it here only as the immediate cause of the appearance of the anasarca, as almost the only cause which is remembered and given on asking the patient how he got his sickness. Blows upon the loins, a hearty draught of cold water, imprudently sitting on a cold stone or upon the damp earth have been referred to as exciting causes.

While thus enumerating the causes it must be remembered that chronic cases can rarely be referred to any one, particular exciting cause. The disease is the result of constitutional predisposition, hereditary, or acquired by the continued operation of one or more of the predisposing causes already named, and almost the first notice the patient has of it, is a bloated face, or hands, or feet, brought on by some chance exposure (see case I, in the June No. of this Journal,). In acute cases on ,the contrary, the predisposing cause can almost always be pointed out, as well as the exciting cause.

